# Adaptive cluster analysis approach for functional localization using magnetoencephalography

**DOI:** 10.3389/fnins.2013.00073

**Published:** 2013-05-14

**Authors:** Hooman Alikhanian, J. Douglas Crawford, Joseph F. X. DeSouza, Douglas O. Cheyne, Gunnar Blohm

**Affiliations:** ^1^Centre for Neuroscience Studies, Queen's UniversityKingston, ON, Canada; ^2^Canadian Action and Perception NetworkToronto, ON, Canada; ^3^Centre for Vision Research, York UniversityToronto, ON, Canada; ^4^Program in Neurosciences and Mental Health, Hospital for Sick ChildrenToronto, ON, Canada

**Keywords:** magnetoencephalography (MEG), cluster analysis, localization of function, machine learning, beamforming

## Abstract

In this paper we propose an agglomerative hierarchical clustering Ward's algorithm in tandem with the Affinity Propagation algorithm to reliably localize active brain regions from magnetoencephalography (MEG) brain signals. Reliable localization of brain areas with MEG has been difficult due to variations in signal strength, and the spatial extent of the reconstructed activity. The proposed approach to resolve this difficulty is based on adaptive clustering on reconstructed beamformer images to find locations that are consistently active across different participants and experimental conditions with high spatial resolution. Using data from a human reaching task, we show that the method allows more accurate and reliable localization from MEG data alone without using functional magnetic resonance imaging (fMRI) or any other imaging techniques.

## 1. Introduction

A number of recent studies have been dedicated to find active brain areas in human reaching/pointing tasks, and to unravel the role of the corresponding regions (Picard and Strick, 2001; Grosbras et al., 2005; Nickel and Seitz, 2005; Blangero et al., 2009; Beurze et al., 2010; Nelson et al., 2010; Vesia et al., 2010). Generally, many brain areas are involved in human reaching tasks. However, to our knowledge, there is no consensus on the areas involved and their role in human reaching tasks. There are some discrepancies between findings from different imaging techniques and/or tasks. This might in part be due to different sensitivities across imaging techniques. Therefore, we believe that it is important to find alternative methods for functional localization of brain regions using previously unexplored techniques, such as MEG. In this study our aim is to propose a method that can reliably be used to consistently find active brain areas from MEG signals.

Recent developments in neuroimaging technologies such as electroencephalography (EEG), magnetoencephalography (MEG), positron emission tomography (PET) and functional magnetic resonance imaging (fMRI) have enabled researchers to localize active brain areas in humans, and also to investigate the role of the corresponding regions. In choosing a neuroimaging method, there has always been a trade off involved between time and spatial resolution. For instance, fMRI has a spatial resolution of millimeters in localizing brain activation though its time resolution is in the order of seconds. On the other hand, MEG has a time resolution of less than milliseconds that enables researchers to investigate brain activity in almost real time. Our aim in this paper is to propose a post processing method on reconstructed MEG spatial filtered signals that can localize brain activity with acceptable spatial resolution, thereby avoiding a duplicate fMRI experiment.

MEG measures magnetic activity in the brain using more than a hundred super conducting sensors on the scalp. The advantage of measuring magnetic signals over electric signals (in EEG for example) is that they can pass the scalp and the tissues underneath without being distorted much because the magnetic permeability of these tissues is approximately the same (as opposed to largely varying electric conductances). For source localization, this constant magnetic permeability is advantageous because it does not need to be measured (Lopes da Silve, 2010).

Although MEG has a high time resolution, localizing active brain areas from the recorded magnetic signals poses a problem. This is by nature an ill-posed problem, and a large body of research has been dedicated to propose solutions to this problem (Gross et al., 2001; Sekihara et al., 2001; Barbati et al., 2004; Cheyne et al., 2006, 2007; Rong and Contreras-Vidal, 2006; Taulu and Simola, 2006; Merrifield et al., 2007).

Adaptive beamforming methods have been proposed to offer robust solutions to the localization problem in MEG studies (Veen et al., 1997; Robinson and Vrba, 1999; Sekihara et al., 2001). The solution is robust in the sense that it can image instantaneous, evoked brain activity even in the presence of large amounts of both environmental noise and intracranially generated artifacts of non-cerebral origin such as from the cardiac muscle, skeletal muscles, and the eyeballs (Lopes da Silve, 2010). In addition, adaptive beamforming does not require specifying the number of interference sources or their forward solutions, making it ideal for MEG data, where both the number and location of brain and interference sources are unknown (Hillebrand and Barnes, 2005).

Beamformer source images can be constructed volumetrically throughout the brain over an arbitrary number of time points for each individual subject and transformed to a common stereotaxic space. A big body of research has been dedicated to improve the performance of the spatial filtering to reconstruct source images (Barnes et al., 2004; Sekihara et al., 2004; Mattout et al., 2007; Woolrich et al., 2011). For instance, in (Barnes et al., 2004) it has been shown that if the sampling of the images is done properly in spatial sampling, beamformer implementations can achieve very high spatial resolution (1–2 mm).

The remaining problem is the post-processing after the source reconstruction to take advantage of this high spatial resolution, namely to determine what brain regions and/or subregions are consistently activated across subjects and experimental conditions (Litvak et al., 2007; Gilbert et al., 2012). In Gilbert et al. (2012), a clustering method has been proposed in which K-means clustering is implemented on a number of peaks that are ranked according to their power for a given number of participants. The goal is to achieve clusters in which peaks represent the same location for different subjects. However, the K-means algorithm does not provide any clue to achieve this goal. On the other hand, in K-means clustering the number of clusters should be given to the algorithm, and there is not any control over the size of the clusters to which the algorithm converges.

To address this problem, we propose an adaptive approach in which a hierarchical clustering algorithm is in tandem with the Affinity Propagation clustering. We show that the proposed method offers a powerful tool to localize brain activity reliably and with acceptable spatial resolution from MEG signals. We use a human reaching/pointing task to localize active brain areas. Note though that the proposed method can be used in any event-related MEG experiment without loss of generality.

## 2. Methods

### 2.1. Participants

Ten healthy adult participants (eight males, two females) age range 22–45 years with no history of neurological dysfunction or injury participated in this study. This study was approved both by the York University and Hospital for Sick Children Ethics Board. All participants gave informed consent.

### 2.2. Experimental paradigm

**Figure 1:**
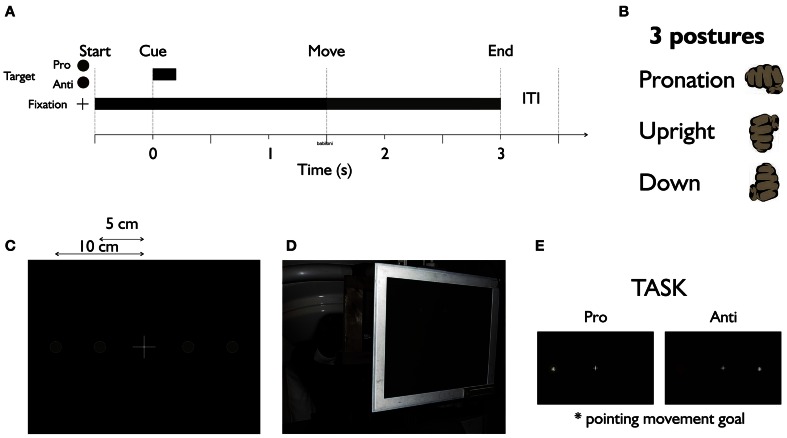
**The MEG experiment setup. (A)** Time course of the experiment. **(B)** Three postures of the hand were used in different recording blocks. **(C)** The fixation cross in the middle with two possible target locations in its left and right hand side. **(D)** Subjects sit upright under MEG machine performing the pointing task with the wrist only. **(E)** Task: target (cue) appears in either green or red to inform the subject of the pro or anti nature of the pointing trials. Dimming of the central fixation cross was the movement instruction for subjects.

Figure 1 shows the experiment setup. Participants were seated in an electromagnetically shielded room with the head under the dewar of an MEG machine while performing memory-guided reaches. Subjects sat upright (Figure 1D), fixating a central white cross. After 500 ms, a green or red dot was briefly presented (200 ms, Figure 1A) randomly right or left of fixation either 5 or 10 cm from the fixation cross (Figure 1C). The color of the dot (red or green, counterbalanced across subjects) indicated the task, i.e., to point toward (pro) or to the mirror opposite location (anti) of the target. Subjects waited for the fixation cross to dim (1500 ms later) before making a wrist-only movement. We used three different forearm/wrist postures, left and right hand (in separate blocks of trials) for pointing (Figure 1B). Each pointing trial lasted approximately 3 s with a 500 ms inter-trial interval (ITI). 100 trials for each condition-left hand versus right hand, pro versus anti, and 3 hand postures-amounts to 1200 trials for each subject.

To make sure that subjects remained fixated we recorded horizontal electro-oculogram (EOG) using temporal electrodes (see below), and we omitted trials where subjects broke fixation.

To measure movement onset for each subject we used bipolar differential electromyography (EMG) with four sets of 3 cm distant electrodes in locations: Exterior Carp: Radialis Longior, Exterior Communis Digitorum, Exterior Carp: Ulnaris, and Superior Longus. Ag/AgCl solid gel Neuroline (Ambu) electrodes of type 715 12-U/C were used for EOG and EMG recordings. EMG and EOG channels were part of the data acquisition system of the CTF MEG system.

### 2.3. MEG data acquisition

We used data acquisition and signal post-processing protocols developed for pre-surgical functioning mapping at the Toronto Hospital for Sick Children. The MEG data was acquired using a 151-channel (axial gradiometers, 5 cm baseline) whole head CTF MEG system (VSM Medtech, Coquitlam, Canada) installed within a magnetically shielded room. Noise levels were below 10fT/$\sqrt{Hz}$ above 1.0 Hz. Prior to MEG data acquisition, each subject was fitted with coils placed at three fiducial landmarks (nasion and pre-auricular points) that were localized by the MEG acquisition hardware to establish the position of the subject's head relative to the MEG sensors.

Structural (*T*_1_-weighted, 3D-SPGR) MRI scans were obtained for each subject using a 1.5 T Signa Advantage System (GE Medical Systems, Milwaukee, WI). Co-registration of the MEG head based coordinate system with the MRI was achieved by identifying the locations of the head localization coils on orthogonal slices of each subject's MRI. For each subject, the inner skull surface was derived from *T*_1_-weighted MR data using the BrainSuite software package (Shattuck and Leahy, 2002).

### 2.4. Data processing

Data were collected at a rate of 600 samples per second with a 150 Hz low pass filter, using synthetic third-order gradiometer noise cancelation. The data was manually inspected for artifacts in addition to eye movements, blinks, premature hand movements and corresponding trials removed from the analysis. On average 98 reaching trials per condition were retained for each subject for subsequent processing.

**Figure 2:**
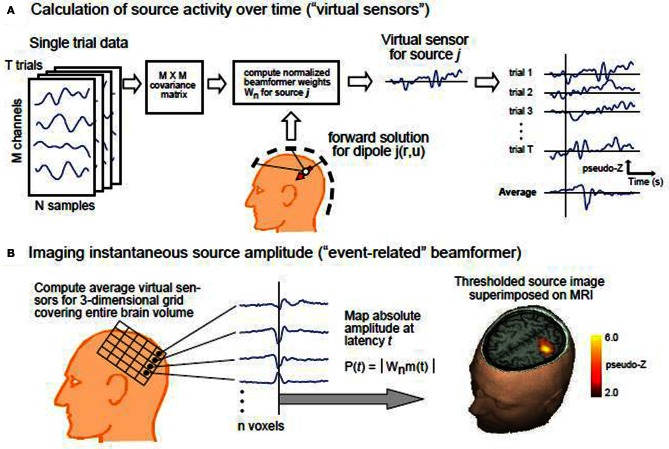
**The diagram of the event related beamformer (Cheyne et al., 2007). (A)** Calculation of source activity over time: the data consists of *T* trials each with *M* channels and *N* time samples. The covariance matrix of the data are given to the beamformer as well as the forward solution for each dipole location. **(B)** Imaging instantaneous source amplitude: Average source activity is then estimated at each voxel, and dipole orientation is adjusted accordingly to maximize power at the corresponding voxel.

We discretized the brain for each subject into 3 mm^3^ voxels, and for each voxel computed the source activity using an event-related beamformer (Cheyne et al., 2006). The beamformer estimates the power at each voxel from the sensor measurements by rejecting the interfering activity from all the adjacent voxels. Because the number of voxels is more than the number of sensor measurements, constraints should be added to the optimization problem to make it mathematically tractable. A typical biologically plausible constraint is to minimize the total variance. Using this method the average or instantaneous power at each voxel can be estimated. A diagram of the beamformer is shown in Figure 2. As is shown in the figure, the event-related beamformer uses the covariance matrix of the measured signal, noise estimate, and forward solution at each voxel to compute the filter weight for the corresponding source location. The covariance matrix is calculated using all the trials for each condition. A noise estimate is provided for the beamformer either using singular value decomposition (SVD) or a constant based on typical white noise levels of the recording environment, thus scaling the output into units of pseudo-Z estimages (Robinson and Vrba, 1999) in order to remove a gain bias of the weights with distance from the sensor array. Estimating signal activity in locations that are presumably more than the number of the channels is an inverse problem which is ill-posed by nature. Therefore, some additional constraints are required to make the problem tractable. Here, the orientation of the dipole is adjusted to maximize power at each voxel.

Average power at each voxel is then computed using the beamformer. At each voxel, we computed average power from 50 to 500 ms after the cue onset and from 200 ms before the movement onset to 350 ms after that. In each case (around the cue onset and movement onset), average power is computed for different frequency bands: 7–15 Hz (alpha), 15–35 Hz (Beta), 35–55 Hz (low gamma), and 55–120 Hz (high gamma).

Different frequency bands have different ranges of detected signal power. Experimentally, lower frequency components typically result in higher power. Using the same threshold across all frequency bands would thus bias results toward lower frequencies. Therefore we used frequency-dependent pseudo-Z score power cutoffs (alpha band: 2; beta-band: 1.5; low gamma: 1; high gamma: 0.5) to extract local maximums from the z-scored average power that is normalized by the estimated SVD noise power. Using this method an average number of 4166± 1288 activation peaks across all subjects have been extracted.

In order to find active areas in the brain during reaching, we merged all the peaks from all the conditions. This results in a 3D image of all the peaks for each subject. The peak coordinates for each subject are then registered from his/her corresponding CTF coordinate to Talairach coordinates using an affine transformation (SPM2, http://www.fil.ion.ucl.ac.uk/spm/).

To find areas that are consistently active in all subjects from the extracted peaks for each subject, we propose to use unsupervised learning algorithms from the machine learning literature. The idea here is to find areas of the brain in which the peaks consistently form clusters. Because the peaks have been put together from all the conditions, the denser they cluster together, the higher would be the chance for the corresponding brain area to become active.

### 2.5. Unsupervised learning

In unsupervised learning, a hidden structure or feature is extracted from the data. This is an optimization problem in which the extracted feature depends on a function to be optimized as well as the set of constraints that are imposed on it. Cluster analysis is a method of unsupervised learning, and in this case the hidden structure that is extracted consists of dividing the input data into separate clusters in a way to optimize a distance measure or a measure of similarity. The choice of the clustering cost function and the optimization algorithm employed to solve the problem determines the resulting clusters (Puzicha et al., 2000; Lashkari and Golland, 2008).

In this study, finding active brain areas can be formulated as an unsupervised learning problem; because there is not any output labeling or structure that we know a prior from the data. In fact it is this structure that we seek to find. In this section we focus on cluster analysis and go over some of the methods that we are using in more detail.

The goal in cluster analysis is to put the objects in separate groups in a way that objects that are in the same group are more similar to each other than objects in other groups. The greater the similarity within a group, and the greater the difference between groups, the more distinct the clustering would become. There are two types of clustering, namely hierarchical clustering, and partitional clustering. While in partitional clustering all clusters are determined at once, in hierarchical clustering, clustering is done successively based on the clusters from the previous stage of the algorithm. Hierarchical clustering can be either bottom–up (agglomerative) or top–down (divisive).

In this paper we solve the clustering problem by proposing the application of the Affinity Propagation algorithm which is a partitional clustering method that unlike the k-means algorithm does not need to know the number of clusters in advance, and converges faster than the k-means algorithm (Frey and Dueck, 2007). As we show in this paper, the Affinity Propagation algorithm can result in clusters that are large for regions with high spatial activity peak density.

We solve this problem by using the Affinity Propagation algorithm as a pre-processing step for an agglomerative hierarchical clustering algorithm. The hierarchical clustering algorithm creates a cluster tree from the data which provides a hierarchy of a similarity measure, i.e., closeness in the Euclidean distance, among data points (average power peaks). Depending on the number of desired clusters and/or the desired cluster size, the tree can be cut at a particular node. Using the Affinity Propagation algorithm as a pre-processing step for the hierarchical clustering has two advantages. First, it reduces the complexity of cluster trees which makes searching in trees for finding a desired node faster. Second, for a computationally efficient clustering, the acceptable size of a cluster should depend on the spatial peak density of a region which itself depends on a particular experiment. Using hierarchical clustering adds a degree of freedom in the clustering process, and can use the spatial density of a region to adaptively determine the coarseness of clustering in the region. The spatial density of the activity peaks can be provided to the hierarchical clustering algorithm by the proposed mesh analysis which discretizes the brain into voxels and computes the normal spatial density in each voxel. Notice that although it is possible to add some constraints on the k-means algorithm like the size of the clusters, the attempt to create clusters with small radii for the large number of activation peaks would cause the algorithm to converge impractically slowly or to not converge at all. Even in the case of convergence, it might end up with many superficial empty clusters that need to be taken care of separately (Pakhira, 2009). Moreover, one might not need to do fine clustering in low spatial density regions. The proposed adaptive computationally more efficient approach addresses these drawbacks by doing finer clustering only on high spatial density clusters.

**Figure 3:**
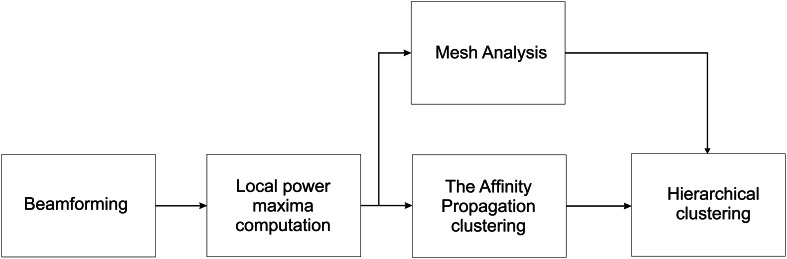
**The flow chart of the proposed method.** The beamformer computes the average power at each voxel. Local maxima of average power is then computed using a frequency-dependent power threshold, and the peaks are extracted. The Affinity Propagation algorithm is used on the resulted peaks to cluster them. A cluster tree is then built by Hierarchical clustering algorithm using Ward's measure as a distance between clusters. The cluster tree is then cut in order to get local smaller clusters of radius 1 cm for regions with normalized spatial density >20%. The normalized spatial density is computed in the mesh analysis block.

Figure 3 summarizes the proposed method. The beamformer computes average power at each brain voxel separately for different frequency bands (alpha, beta, low gamma, high gamma) and different conditions. The combination of right-hand/left-hand, wrist posture (pronation, upright, down) with pro/anti movement is considered as one condition. For each condition and frequency band peak maxima are then calculated according to the frequency dependent power thresholds. The peaks from all the conditions and frequency bands are then put together to create data input for the Affinity Propagation clustering and mesh analysis blocks. The resulting clusters (from the Affinity Propagation algorithm) as well as normalized spatial density of the peaks (from the mesh analysis) are given to the hierarchical clustering algorithm as inputs.

Cluster trees (dendrograms) are then generated using Ward's linkage for clusters with normalized spatial density greater than 20%. This threshold can be adjusted based on individual data sets. The goal in this analysis is to use the proposed method to find areas that are consistently active among all the conditions for each subject as well as among all subjects. we found that for densities less than 20%, peaks were not consistent among different experimental conditions. Therefore, 20% threshold is a good trade-off between reliability and detail for this reaching experiment.

The trees are then cut to find clusters with 1 cm radius. If the standard deviation of peak distances from their corresponding centroids at each cluster location is less than 1 cm among all subjects, we call the corresponding brain area active. The standard deviations are reported for the areas that we found active in Tables 2, 3. Notice that the choice of 1 cm which can be adapted to individual experiments puts an upper bound on the localization precision while the lower bound is identified by the beamformer discretization level.

Algorithm 2.1 is the pseudo code for the proposed method in which the proposed method is explained with suggestions on the functions that can be used to implement it. Notice in the algorithm that activation peaks are extracted for each frequency band separately (using the CTF SAM toolbox, MEG International Services Ltd., Coquitlam, BC, Canada), with separate thresholds (more detail on the thresholds is the section 2.2). The extracted peaks are then put together to be clustered by the Affinity Propagation algorithm. Mesh analysis calculates the normalized spatial density of the peaks which is given to the hierarchical clustering in order for that to perform finer clustering in areas with normalized spatial density of greater than a given threshold, e.g., >20%.

**Algorithm 2.1:**
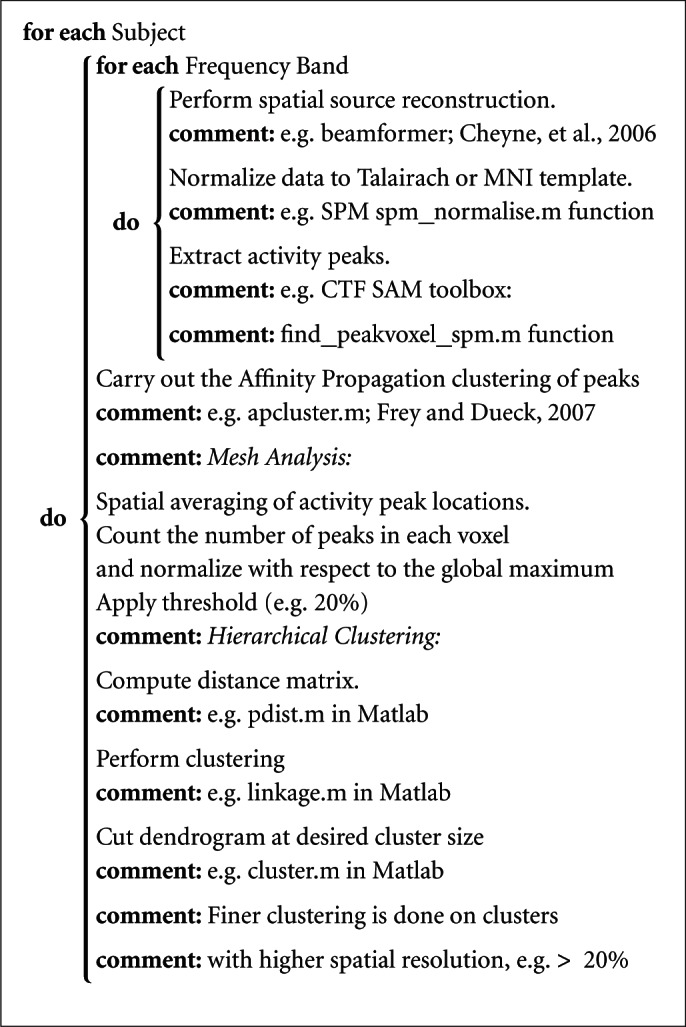
Adaptive Clustering()

#### 2.5.1. Affinity propagation algorithm

The Affinity Propagation algorithm (Frey and Dueck, 2007) is an unsupervised learning method based on message passing between data objects. The objective of the message passing is to identify a subset of representative examples from the data that minimize a total similarity objective function. These representative examples are called exemplars (or cluster centers).

The algorithm uses a similarity matrix as input that captures the similarity between any two pairs of objects that are to be clustered. Depending on the application, different measures can be chosen as similarity criteria, and there is no unique choice for that. In the case of our study we are using negative Euclidean distance as a similarity measure. Thus, the closer the peaks are to one another, the more similar they are.

The Affinity Propagation algorithm does not need to know the number of clusters *a priori*, and indeed this is one of the advantages of this technique. Instead, one only needs to specify the diagonal elements of the similarity matrix (similarity of each peak to itself), and by specifying the greater diagonal number for a peak, the higher priority is given to that peak to become an exemplar. To provide the same chance for all the extracted peaks to become exemplars, all diagonal elements are given the same quantities. In our study, we choose the median of similarities as diagonal elements to make sure that diagonal entries of the similarity matrix are in the same range as the rest of the entries (Frey and Dueck, 2007).

Two kinds of messages are exchanged in this algorithm namely, responsibility and availability. The responsibility message *r*(*i*, *k*) sent from peak *i* to the exemplar candidate peak *k* reflects the accumulated evidence for how well-suited peak *k* is to serve as exemplar for peak *i*, taking into account other potential exemplars for peak *i* (Frey and Dueck, 2007). The availability *a*(*i*, *k*) sent from peak *k* to peak *i*, as the name suggests, shows the amount of availability of peak *k* to be chosen as an exemplar by peak *i*, taking into account the support from other peaks for which the peak *k* should be an exemplar (Frey and Dueck, 2007).

In this study, we chose the damping factor λ of the algorithm to be 0.5. The choice for this parameter is not unique. However, it should not be chosen too large (close to one) preventing the algorithm from converging or too small (close to zero) slowing down the convergence speed. We stopped the algorithm when the amount of change in the messages falls below 1%.

For the implementation, we used the m-file “apcluster.m” that is provided by Dr. B.J. Frey and D. Dueck in their website: http://www.psi.toronto.edu/affinitypropagation/software/apcluster.m.

#### 2.5.2. Hierarchical clustering

In agglomerative clustering the first stage starts with the assumption that each of the objects forms a cluster just by themselves. So the number of clusters is equal to the number of objects. Then at each stage two of the clusters that are most similar to one another merge and form a new bigger cluster. This procedure continues to the stage where all the objects form one cluster together. The solution to the problem in hierarchical clustering is a tree called dendrogram with objects as its nodes, and edges as representations of the objects that are merged together at each step. The advantage of this type of clustering is that the number of the clusters needs not to be known in advance. The tree can be cut at any stage to get the clusters, and depending on the stage of the cut, different numbers of clusters would emerge.

In hierarchical clustering, it is crucial to define a similarity function between any two clusters, and depending on this definition a variety of hierarchical clustering techniques can be defined.

In this paper we use Ward's (Ward, 1963) method as a measure of distance between clusters (Matlab linkage.m function is used, The MathWorks, Natick, MA, USA). Ward's method uses the increase in the total within-cluster sum of squares as a result of joining clusters *a* and *b*. The within-cluster sum of squares is defined as the sum of the squares of the distances between all objects in the cluster and the centroid of the cluster.

The advantage of using Ward's criterion is that because of its incremental design in the definition of distance, it produces a cluster tree that is monotonic. A cluster tree is not monotonic when sections of the dendrogram change direction. This occurs when the distance from the union of two clusters *a* and *b* to a third cluster *c* is less than the distance from either the distance from *a* to *c* or the distance from *b* to *c*, i.e., the triangle inequality is not satisfied. This non-monotonic cluster tree can occur when, for instance, the distance between clusters is defined as a distance between their centroids. Moreover, Ward's linkage is biased toward producing compact clusters with approximately the same number of observations rather than the complete linkage that produces clusters with approximately equal diameters which might not be as compact (Milligan, 1980). Clusters with the same number of observations make the cross-validation of the resulting clusters easier among subjects. It has also been shown (Milligan, 1980) that variance based methods (like Ward's) perform better than the complete linkage.

When two clusters are equidistant from a third cluster non-uniqueness in dendrogram cuts results in the ties-in-proximity problem. In this study, we are not dealing with integer valued or binary data for which there is a greater chance of running into this problem (Fernandez and Gomez, 2008). In addition, Ward's linkage is less susceptible to this problem than simple or complete linkages (Fernandez and Gomez, 2008). Finally, for each implementation of the hierarchical clustering, rather than implementing the clustering on the whole brain, we reduce data set size by focusing on a cluster with high spatial density. This reduction in the data set size not only reduces the complexity of the resulting dendrograms, but also reduces the chance of running into the ties-in-proximity problem (Fernandez and Gomez, 2008).

Hierarchical clustering yields clustering algorithms that avoid the difficulty of trying to solve a hard combinatorial optimization problem, i.e., it cannot be viewed as globally optimizing an objective function. Because of this fact, these algorithms do not have problems with local minima or difficulties in choosing initial points. These algorithms tend to make good local decisions about combining two clusters since they have the entire proximity matrix available. However, once a decision is made to merge clusters, the scheme does not allow for that decision to be changed. This prevents a local optimization criterion from becoming a global optimization one (Tan et al., 2005).

### 2.6. Mesh analysis

To compute the spatial density of the clusters, we discritized the brain into 1 mm cubes, counted the number of peaks in each cube, and normalized the numbers with respect to the cube with the largest number of peaks to come up with a normalized spatial density of the peaks in the brain for each subject individually. To get a smooth spatial density image and to reduce the dependence of the spatial density to the discritization level, the cube size should be chosen as small as possible, but not too small to make the processing computationally slow. We found that 1 mm cube-size meets this trade off fairly well. In the rest of the paper, we refer to this method as mesh analysis.

## 3. Results

**Figure 4:**
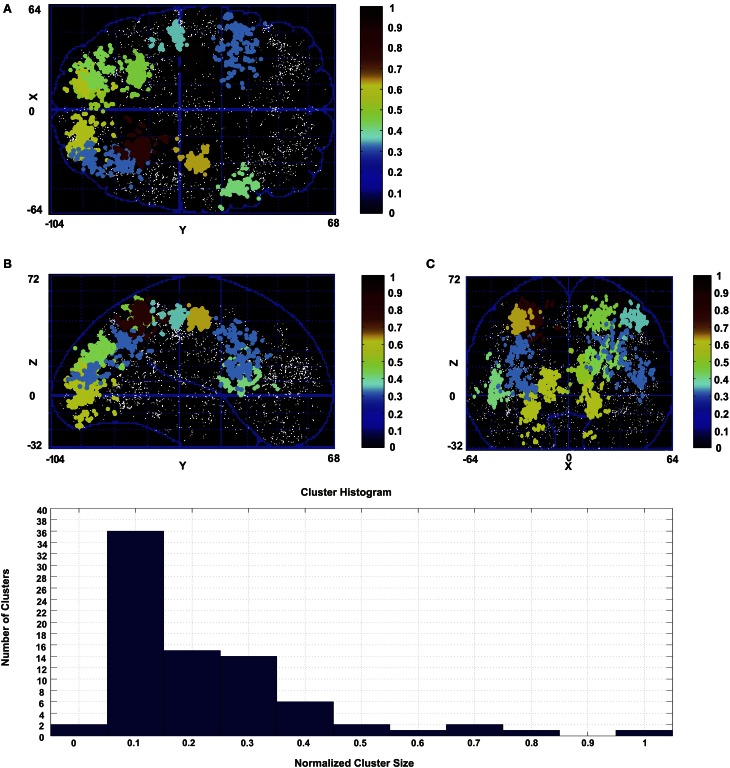
**Clusters emerged from the Affinity Propagation algorithm for subject 1.** Only the clusters with the number of peaks greater than 28.5% of the biggest cluster (397 peaks) are shown. Clusters are color coded according to their number of peaks with respect to the biggest cluster. **(A)** Transverse view in Talairach coordinates. **(B)** Sagittal view in Talairach coordinates. **(C)** Coronal view in Talairach coordinates. The histogram of the cluster sizes is shown at the bottom.

As mentioned, for the K-means algorithm the number of clusters should be known in advance. Because we do not know the number of clusters, we use the Affinity Propagation algorithm instead. Figure 4 shows the result of the Affinity Propagation clustering for subject 1. In the Affinity Propagation algorithm we used the negative square of the Euclidean distance as a similarity measure. For self similarity, i.e., the elements on the diagonal of the similarity matrix, we used the median of similarities. Thus, we gave all the peaks the same chance to become exemplars. We chose the damping factor λ=0.5.

On average 58± 12 clusters have been found across all subjects by the algorithm. In the figure the clusters are color coded depending on their number of peaks from blue for the smallest to red for the biggest. In Figure 4 not all the clusters are shown for the subject. In subject 1 the size of the biggest cluster is 397, and this cluster is shown with the red color. Only the clusters with more than 28.5% of the size of the biggest cluster are shown for this subject. Choosing this number for the figure was merely for illustration purpose. A smaller number renders the figure too crowded to be illustrative. We choose a small enough percentage to show a significant number of clusters without overcharging the figure. The histogram of the number of clusters versus the normalized cluster sizes for this subject is shown at the bottom of the figure.

As is evident from the figure, we found that many areas mainly from occipital and parietal regions are involved in the human reaching task. In almost all the subjects the biggest cluster is associated with pre-motor and primary motor areas followed by visual areas and a large network of parietal regions. Some clusters have also been found in frontal areas. Clusters in this region are bigger in physical size and more sparse with less active peaks. For this reason they are not included in Figure 4.

**Figure 5:**
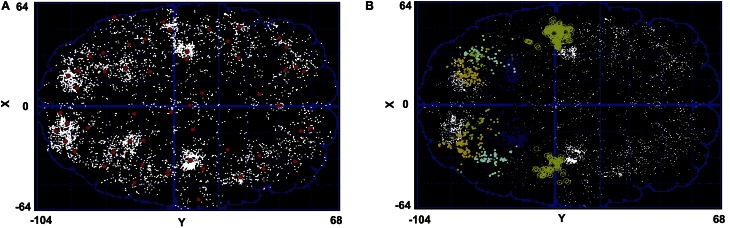
**(A)** Cluster centers (red dots) resulted from the Affinity Propagation algorithm for subject 1. All the peaks have been given the same chance to become exemplars. White dots are the regions with spatial peak density >10%. **(B)** Parietal clusters in both hemispheres. Some clusters in parietal region are so big in their physical size that they can not be attributed to only one brain region.

More specifically, we look at distinguishably different parietal regions. Figure 5A shows the centers of all the clusters for subject 1. Figure 5B shows all of the parietal clusters in both hemispheres for this subject. As is evident from this figure, some of the clusters that have been found by the Affinity Propagation algorithm are so physically big that they can not be attributed to only one brain area. In meta analysis literature the standard deviation of a region is in the order of millimeters for each dimension (Nickel and Seitz, 2005; Mayka et al., 2006; Blangero et al., 2009; Vesia et al., 2010). This order of standard deviation makes the volume of the cluster associated with a corresponding region in the order of cm^3^ at most.

**Figure 6:**
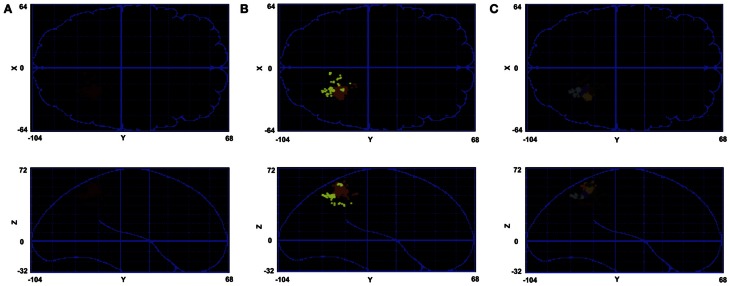
**An example of the proposed method. (A)** A cluster in parietal area resulted from affinity propagation algorithm (radius: 22 mm). **(B)** The effect of cutting the cluster tree that leads to two clusters (maximum radius: 11 mm). **(C)** The effect of cutting the cluster tree that leads to seven clusters (maximum radius: 4 mm).

To see the problem more specifically, Figure 6A highlights the biggest cluster-in terms of the number of the constituent peaks-in the parietal area of subject 1. The region has a physical volume of approximately 8000 (mm)^3^. On the other hand, such a big volume of active peaks can not be seen in other subjects. Thus, this big cluster might have emerged by merging more than one active clusters, and can not be attributed to one parietal region.

To solve this problem of the Affinity Propagation algorithm, i.e., to find active areas with higher spatial accuracy and more consistency among the subjects, we propose the use of hierarchical clustering in tandem with the Affinity Propagation. As it was mentioned in the section 2, hierarchical clustering provides us with a clustering tree called dendrogram with the end leafs and the top node corresponding to the peaks as individual clusters and all the peaks as one cluster, respectively. The advantage of this clustering method is that the tree can be cut at different stages to provide us with clusters of desirable sizes.

On the other hand, using hierarchical clustering alone for large data sets is not computationally efficient. The large total number of peaks for each subject makes implementing hierarchical clustering result in a huge tree. Searching in the tree in order to find a cutting point that results in clusters with acceptable physical size requires huge amount of trial and error, and is not computationally efficient. In general, hierarchical clustering algorithms are not computationally efficient when the number of objects to be clustered is big (the order of 1000) like the case we are dealing with here.

To tackle this problem, we propose to use Affinity Propagation for clustering in the first step. Then, hierarchical clustering can be done on the clusters that have been found by the Affinity Propagation algorithm. These clusters are considerably smaller in their number of points than all of the active peaks, and the location of the clusters are known. So, we are dealing with smaller cluster trees and we also know in what particular region we are doing hierarchical clustering. Depending on the desired spatial resolution in a particular region, the corresponding tree can be cut. For each of the clusters that is found using the Affinity Propagation algorithm, we used hierarchical clustering, and we cut the trees in a way to find sub-clusters of radius less than 1 cm. The resulting clusters with this radius and more than one peak are considered as active clusters when the standard deviation of the distances from the peaks to their corresponding centroids across all subjects is less than 1 cm as well.

Other partitional clustering algorithms like the k-means can be used as the first step as well. However, the number of clusters should be given to the algorithm, and finding such a number would require some trial and error and is a computational burden. Our analysis for this data set reveals that 40–50 clusters is a good estimate to be given to the k-means algorithm depending on the subject.

**Figure 7:**
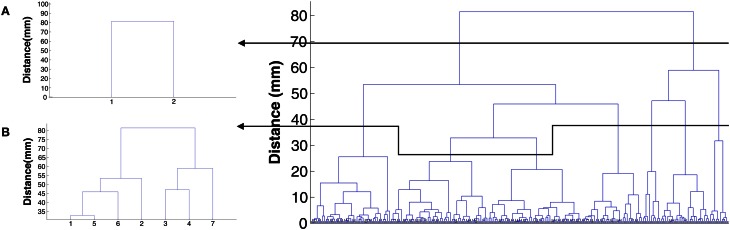
**Cluster tree for the cluster in Figure 6A. (A)** The cut that leads to Figure 6B. **(B)** The cut that leads to Figure 6C.

Figures 6B,C show the effect of hierarchical clustering on the cluster in Figure 6A. In these figures the tree is cut in a way that leads to two and seven smaller clusters, respectively. Figure 7 shows the dendrogram of Figure 6A with the cuts that lead to Figures 6B,C clusters.

**Figure 8:**
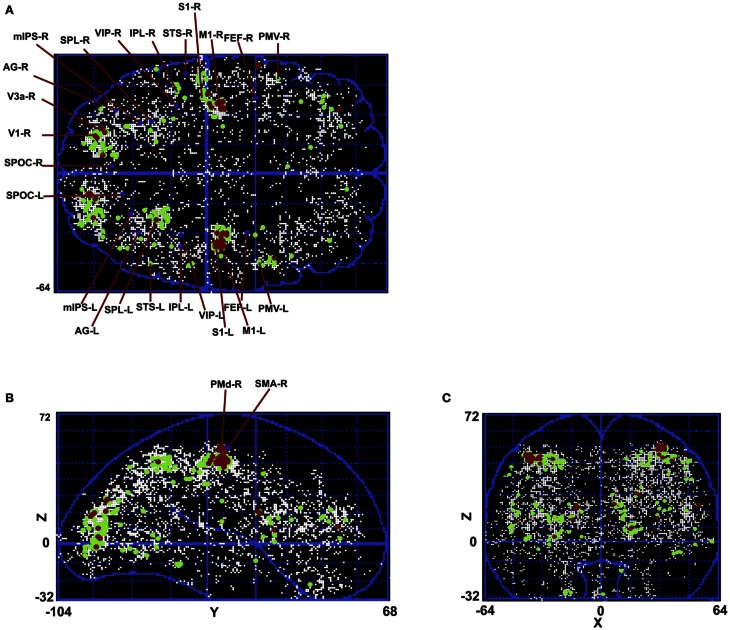
**Mesh analysis method is used to find normalized spatial density.** Areas with spatial density from 10 to 20%, 20 to 30%, and greater than 30% are shown in white, green, and red, respectively. **(A)** Transverse view in Talairach coordinates. **(B)** Sagittal view in Talairach coordinates. **(C)** Coronal view in Talairach coordinates.

Figure 8 shows the calculated density (mesh analysis) for subject 1. In the figure, areas with density from 10 to 20%, 20 to 30%, and greater than 30% are shown in white, green, and red, respectively. The aim of this analysis was to study cluster densities in different brain regions. In addition, normalized spatial density is given to the hierarchical clustering algorithm as the second input, providing the algorithm with the information to find the appropriate tree cut. All the active areas that are found using cluster analysis for this subject are shown in the figure. The highest density of activation is located in occipital and pre-frontal areas with highest spatial density in pre-motor, motor, sensorimotor, and visual cortical areas V1 and V3 that are shown in red. A big network of parietal areas is also shown with fairly high spatial density in green including superior parietal lobule (SPL), inferior parietal lobule (IPL), and ventral intra-parietal area (VIP). Frontal eye field (FEF) is also active in all subjects in the left hemisphere.

Table 1 summarizes the areas that we have found consistently active in all subjects. The areas that have spatial density of more than 30% using the mesh analysis are highlighted in gray. Active and non-active areas are shown in black and red dots for each subject, respectively. When the standard deviation of distances across all subjects (for which clusters of radius <1 cm could be found) in a region is less than 1 cm (the amount that was chosen for tree cuts), we called the corresponding region active and we included that in the table as black dots for the corresponding subjects. In some subjects, however, no cluster of radius <1 cm could be found in some regions which are shown as red dots in the table.

**Table 1:**
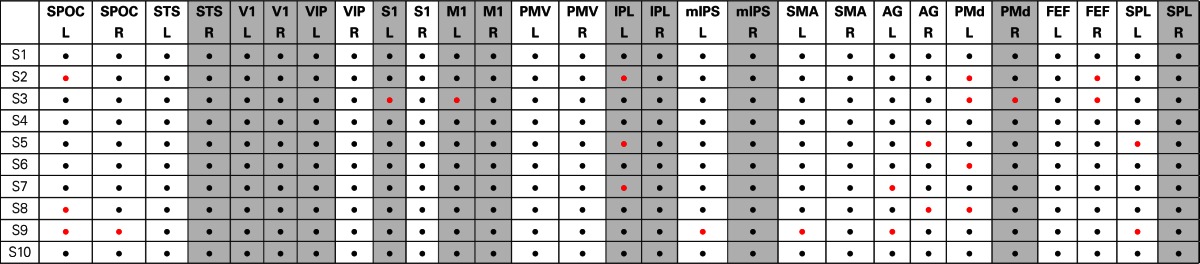
**Summary of the active brain areas in human reaching task using cluster analysis**.

*The highlighted columns are the areas with spatial density of more than 30%. Active and non-active areas are shown in black and red dots for each subject, respectively*.

*SPOC, superior parieto-occipital cortex; STS, superior temporal sulcus; V1, primary visual cortex; VIP, ventral intra-parietal area; M1, primary motor cortex; S1, somatosensory cortex; PMV, ventral pre-motor cortex; PMd, dorsal pre-motor cortex; IPL, inferior parietal lobule; mIPS, mid-posterior intra-parietal sulcus; SMA, supplementary motor area; AG, angular gyrus; FEF, frontal eye field; SPL, superior parietal lobule*.

Tables 2, 3 show the mean locations and standard deviations of the areas that we found active in the left and right hemisphere, respectively. For comparison, Table 4 summarizes the mean locations of the same regions that are reported by the fMRI literature. Comparing the locations that are reported in the fMRI literature (Table 4) with the mean locations that we have found (Tables 2, 3) shows that the locations of the areas that we found active in this MEG study are in close agreement with fMRI literature.

**Table 2:** **Mean location and standard variation of the regions across all 10 subjects for the left hemisphere**.

**Area**	**Mean location (mm) (Talairach coordinates)**			**Standard deviation (mm)**		
	***x***	***y***	***z***	***x***	***y***	***z***
Left hemisphere						
STS	−45.2	−57.1	14.7	4.2	2.6	3.7
PMV	−49.6	4.8	21.3	3.0	3.0	3.8
IPL	−42.5	−35.3	49.2	3.9	4.9	3.4
VIP	−37.1	−40.2	44.4	2.4	3.5	2.3
FEF	−28.4	−1.2	43.4	4.4	4.2	3.6
SPL	−23.2	−54.3	46.0	3.8	3.8	6.7
mIPS	−22.0	−61.3	39.5	2.0	3.5	4.6
M1	−35.1	−23.4	53.8	4.4	4.4	3.8
S1	−39.5	−24.5	48.0	3.7	2.9	3.5
AG	−35.3	−60.8	35.4	4.9	7.5	4.0
SMA	−4.4	−9.2	51.8	4.6	6.5	2.9
SPOC	−9.0	−71.0	36.7	6.7	4.8	5.8
PMd	−30.0	−1.4	47.0	8.3	5.4	8.2

**Table 3:** **Mean location and standard variation of the regions across all 10 subjects for the right hemisphere**.

**Area**	**Mean location (mm) (Talairach coordinates)**			**Standard deviation (mm)**		
	***x***	***y***	***z***	***x***	***y***	***z***
Right hemisphere						
STS	48.5	−40.5	11.7	2.9	4.9	4.6
PMV	48.9	8.4	21.2	4.2	3.8	3.3
IPL	40.9	−40.6	39.3	3.7	4.6	4.6
VIP	37.0	−44.0	47.3	3.9	2.6	5.1
FEF	31.2	−2.2	44.7	5.1	5.3	6.6
SPL	26.9	−55.1	49.3	4.8	2.2	2.3
mIPS	23.3	−61.8	40.4	4.1	4.5	5.6
M1	36.7	−23.0	52.4	3.3	5.0	4.2
S1	39.2	−25.9	40.2	3.8	4.7	25.3
AG	32.2	−69.5	34.7	4.2	4.7	2.6
SMA	2.7	−7.0	48.9	3.3	4.9	3.6
SPOC	9.6	−77.0	34.4	8.1	3.1	4.8
PMd	28.6	−5.3	49.9	3.9	8.0	6.2

**Table 4:** **Mean locations in Talairach coordinates that are reported in the literature**.

**Area**	**Mean location (mm) (Talairach coordinates)**			**References**	**Distance (mm)**
	***x***	***y***	***z***		
STS	50	−40	12	Grosbras et al., 2005	1.61
PMV	−50	5	22	Mayka et al., 2006	0.83
IPL	−32	−40	52	Blangero et al., 2009	11.84
VIP	38	−44	46	Bremmer et al., 2001	1.64
FEF	31	−2	47	Paus, 1996	2.32
SPL	23	−64	44	Nickel and Seitz, 2005	11.07
mIPS	18	−60	54	Blangero et al., 2009	14.71
M1	−37	−21	58	Mayka et al., 2006	5.20
S1	−40	−24	50	Mayka et al., 2006	2.12
AG	36.3	−70.5	42.6	Vesia et al., 2010	8.97
SMA	−2	−7	55	Mayka et al., 2006	4.57
SPOC	9.5	−80.6	44.2	Vesia et al., 2010	10.44
PMd	−30	−4	58	Mayka et al., 2006	11.30

*The last column shows the distances from the corresponding mean locations that are found from the MEG analysis*.

In addition, spatial resolution that is revealed by the standard deviations of the order of millimeter (except for right S1 z-coordinate: Table 3) is comparable with the high spatial resolution of fMRI. This high spatial resolution that is achievable using the proposed method on MEG signals confirms that the method can be reliably used to localize brain areas in MEG experiments without the need to do a duplicate fMRI experiment.

## 4. Discussion

In this study we proposed adaptive clustering approach to localize brain activity using MEG accurately and reliably to address MEG localization difficulties due to large variations in signal strength, and the spatial extend of the reconstructed activity. With the proposed method it is possible to take advantage of the high time resolution in MEG after finding the active areas. The limitations that are imposed by the MRI machine on the experiments are avoided as well.

The method takes advantage of the recent spatial filtering advances to solve the inverse problem of finding activation peaks in a discretized brain from MEG measurements (Cheyne et al., 2007). To include different ranges of detected signal power in different frequency bands, a frequency-dependent power threshold is proposed to extract local power maxima. In a second step the Affinity Propagation algorithm is proposed to cluster the resulted peaks. Third, we suggest using a top–down hierarchical clustering method based on Ward's measure in tandem with partitional clustering to localize brain regions with higher spatial resolution. Depending on the spatial resolution needed, hierarchical cluster trees can be cut to obtain the desirable standard deviation.

MEG resolution is paradigm/brain area/frequency dependent. As a result, desired cluster sizes should be adapted accordingly. Putting hierarchical clustering that is provided with normalized spatial density of extracted peaks in tandem with the Affinity Propagation algorithm creates this adaptability by enabling finer clustering in areas with higher spatial density.

The analysis is based on clustering power peak locations that are measured across different conditions/repetitions. We have shown that this leads to very reliable localization of specific brain areas with sub-centimeter resolution for all our subjects independently. Notice that this does not mean that signal power in the brain is localized with high spatial resolution; it simply means that one can get reliable estimates of the peak location of a certain brain area regardless of the spatial extent of raw beamformer results. This is due to the presence of many independently extracted peak locations (across different conditions/trials) that appear to be quite reliably localizable.

The areas that have been found using our proposed method in the human reaching task are in agreement with meta analysis literature (Paus, 1996; Bremmer et al., 2001; Grosbras et al., 2005; Nickel and Seitz, 2005; Mayka et al., 2006; Blangero et al., 2009; Vesia et al., 2010). On the other hand, to our knowledge this is the first study that is looking at all the areas involved in the human reaching task using MEG data.

In this study we are extracting the activation peaks merged for all the conditions in the experiment. We have not focused on a particular frequency band or time moment either. The goal here was to investigate all the areas that are involved in a human reaching task no matter in which condition. This is the reason why extracted peaks from all conditions are merged together before cluster analysis. Therefore, some regions might be selectively active during certain conditions, but analyzing these details is beyond the scope of this study.

The average power within different frequency bands and in a 500 ms window was used to extract the peaks to capture the main activations during the pointing task. However, technically it is possible to extract the instantaneous power, and find local maxima with the same procedure. Performing the proposed cluster analysis on the resulted peaks would lead to instantaneous activation patterns. Because of the MEG millisecond time resolution, active brain areas can be found at exact time points with spatial resolution comparable to fMRI. In addition, if the beamformer power estimates are band-pass filtered before doing the cluster analysis, activation areas can be found for different frequency bands. Therefore, the proposed method is also useful in extracting active areas for a particular condition or frequency band. To that end, frequency/time limitations should be imposed on the beamformer solution for the corresponding condition. Once the inverse problem is solved by the beamformer and local peaks are extracted, the proposed method can be used to extract the active areas as well.

It has been shown in (Barnes et al., 2004) that if sampling is done with large enough rate, beamformers can achieve high spatial resolution in the order of millimeters. Because the proposed method is based on cluster analysis on the reconstructed source data, theoretically it can not achieve better spatial resolution than the beamformer.

Head movement is a potential cause of error in the analysis. In this study, head position was measured at the beginning and end of each block of trials and data with head movements >5 mm have been removed. Moreover, we did not merge the extracted activity peaks across subjects to do the clustering. This was done precisely to avoid inter-subject differences and distortions induced by spatial transformations. Rather, we carried out the clustering for each subject separately to make such errors independent from each other.

Because the proposed cluster analysis is done on MEG measurements, it inevitably carries MEG measurement limitations. In a spherical conductor fields generated by impressed currents and volume currents cancel each other out but only for radially oriented currents. Thus, the activity in gyri is hard to detect while sulci activity is relatively easier to detect.

On the other hand, the generated magnetic field is perpendicular to the corresponding electric field. Thus, for the regions for which magnetic signals are hard to detect, electrical activity can be measured easier and more exactly (Babiloni et al., 2004). Therefore, for finding brain areas more accurately it would be better if EEG was measured simultaneously with MEG. Indeed, because EEG and MEG are complimentary it might even be of interest to combine activity peaks measured from both techniques.

The number of extracted activation peaks is also important. Technically the proposed method can be implemented on any number of peaks. But care should be taken in interpreting the results if the number of peaks is small. To make sure that the number of peaks is large enough, in this study we computed source activity for voxels with volumes as small as 3(mm)^3^. In addition, we used a conservative frequency-dependent power threshold to extract local power maxima. This led to 2500 peaks on average for each subject.

While choosing a small number of peaks leads to a coarse spatial resolution, choosing a large data set makes the problem computationally difficult to solve. To address this trade off more wisely, it would be possible to choose the number of peaks differently for different brain regions based on a pre-knowledge of the brain activity for a specific experiment. For instance, our mesh analysis results show that for reaching/pointing tasks the spatial peak density is higher for occipital and parietal areas and much lower in frontal areas. Therefore, it is possible to choose a large number of peaks (or smaller voxel volumes) for occipital and parietal regions and relatively smaller number for frontal regions in reaching/pointing experiments.

### Conflict of interest statement

The authors declare that the research was conducted in the absence of any commercial or financial relationships that could be construed as a potential conflict of interest.
